# Assessment of Dry Eye Syndrome Among Contact Lens Users in Asir Region, Saudi Arabia

**DOI:** 10.7759/cureus.21526

**Published:** 2022-01-23

**Authors:** Abdulrahman Alamri, Khaled A Amer, Abdulrahman A Aldosari, Sereen D Al-Muhsin, Razan S Al-Maalwi, Shahd A Al Hamdan, Lujane M Al-Tarish

**Affiliations:** 1 College of Medicine, King Khalid University, Abha, SAU

**Keywords:** saudi arabia, effect, risk factors, relations, dry eye, wearers, users, contact lenses

## Abstract

Background

Patients with contact lens-associated dry eye (CLADE) disease had different symptoms, such as dryness, foreign body sensation, eye strain, and blurred vision. In addition, CLADE is characterized by end-of-day pain. The degradation of the ocular surface as a result of extended dryness or employment with a visual display terminal (VDT) causes these symptoms. Because these symptoms worsen as the day progresses, practitioners should evaluate contact lens wearers at the end of the day to identify symptomatic patients.

Aim

To assess dry eye syndrome among contact lens users in the Asir region, Saudi Arabia.

Methodology

A descriptive cross-sectional study was used, targeting all accessible contact lens users in the Asir region, South of Saudi Arabia. A total of 250 individuals received the study survey. Data were collected from participants using an online pre-structured questionnaire. The authors initiated the study tool with the help of a comprehensive review of similar articles in the literature and consulting specialized experts, including an ophthalmologist and Arabic translator, to ensure the accuracy of the questionnaire's translation process. The questionnaire covered the following data: participants' socio-demographic data, contact lens use and frequency of use, dry eyes symptoms (DESs) questions, Contact Lens Dry Eye Questionnaire-8 (CLDEQ-8), and Ocular Surface Disease Index (OSDI).

Results

Out of 405 respondents, only 201 contact lens users in the Asir region were included in the study, with a participation rate of 80.4%. Respondents' ages ranged from 18 to 50 years with a mean age of 24.3 ± 12.8 years old. A total of 122 (60.7%) contact lens users had eye disease, including myopia among 94 (77%), hyperopia among 8 (6.6%), and 20 (16.4%) had other eye diseases. A total of 91% of the study participants felt dry eyes two weeks before study time, 88.1% experienced burning sensation, and 82.6% complained of red-eye. A total of 131 (65.2%) contact lens users had non-dry eyes, 41 (20.4%) had mild dry eyes, 20 (10%) had moderate dry eyes, and 9 (4.5%) had severe dry eyes. A total of 48.8% of those who used lenses daily had dry eyes compared to 25% of those who used lenses annually (P = 0.049).

Conclusions

In conclusion, the current study showed that nearly one out of three contact lens users in the Asir region experienced contact lens-related dry eye syndrome mainly with a moderate degree. A higher dry eye syndrome rate is estimated among those who reported a higher frequency of contact lens use and others with a history of eye diseases.

## Introduction

Dry eye disease is a rising public health challenge associated with visual uneasiness, exhaustion, and visual disorders that badly affect the quality of life, including somatic, social, and mental sectors, daily activities, and workplace productivity [1-4]. The physical effect of the dry eye appears to be mainly associated with long-term pain, which causes persistent ocular surface irritation and subsequently will have a negative impact on quality of life [5].

Contact lens users persistently experience discomforts. The Tear Film and Ocular Surface Society (TFOS) International Workshop on contact lens discomfort (CLD) reported the correlation between contact lenses and ocular health [6]. Eye blinking plays an essential role in cleaning the eye's surface of any debris and washing it out with fresh tears. Staining on the eyelid margin is a clinical condition known as lid wiper epitheliopathy (LWE), which is observed to be one of the consequences of incomplete blinking [7]. Among soft contact lens users, there is insufficient evidence regarding incomplete blinking. Though, many findings estimated the relation between corneal fluorescein staining score and the amount of inadequate blink among soft contact lens users [8-10]. Furthermore, soft contact lens wearers with incomplete blinking tend to show CLADE disease [9].

Patients with CLADE disease show different clinical presentations, including dryness, eye redness, foreign body sensation, eye strain, and blurred vision. The symptoms are worse in the evening/night than during the day. These symptoms are caused by the worsening of the ocular surface due to long period dryness or visual display terminal (VDT) work. In patients with dry eye disease, impaired visual acuity has been reported using functional visual acuity that helps to measure dynamic changes of visual acuity [11-13]. The current study aimed to assess dry eyes syndrome among contact lens users in the Asir region.

## Materials and methods

A descriptive cross-sectional study was used, targeting all accessible contact lens users in the Asir region, south of Saudi Arabia. The Research Ethics Committee approved the study at King Khalid University with approval number (ECM#2021-5707). A total of 405 individuals received and filled out the study survey. Patients under 18 years of age and who had a previous history of eye surgery or had ocular infection were excluded. A total of 201 respondents met the inclusion criteria of the study with a participation rate of 80.4%. Data were collected from participants using an online pre-structured questionnaire. The researchers initiated the study questionnaire after a complete literature review and expert consultation in the study's field issue. The study questionnaire was reviewed using a panel of three experts for validity and clarity. The questionnaire covered the following data in four sections: the first section included participants' socio-demographic data like age, history of eye diseases, contact lens use, and frequency of use. The second section included dry eyes symptoms (DESs) questions. The third part included Contact Lens Dry Eye Questionnaire-8 (CLDEQ-8), and the last section was Ocular Surface Disease Index (OSDI). Tool reliability was assessed using a pilot study of 25 participants with a reliability coefficient (α-Cronbach's) of 0.71 for OSDI and 0.76 for CLDEQ-8 items. The questionnaire was uploaded and distributed online using social media platforms and WhatsApp from 20 September 2021 to 20 November 2021. All accessible and eligible lens users in the study setting were invited to fill out the attached tool.

Data analysis

After data were extracted, it was revised, coded, and fed to statistical analysis software IBM SPSS version 22 (SPSS, Inc. Chicago, IL). All statistical analyses were done using two-tailed tests. P-value less than 0.05 was statistically significant. Dry eye was assessed based on OSDI where an OSDI score of 0-12 represents a non-dry eye; an OSDI score of 13-22 represents a mild dry eye; an OSDI score of 23-32 represents a moderate dry eye; an OSDI score of >32 represents a severe dry eye [14]. Descriptive analysis based on the frequency and percent distribution was done for all variables, including contact lens user's bio-demographic data, lens use frequency, and history of eye diseases. Also, frequency distribution for DESs questions and CLDEQ-8 was tabulated. Cross-tabulation was used to assess the distribution of contact lens users' dry eyes by their bio-demographic data and contact lens use frequency. Relations were tested using Pearson's chi-squared test and exact probability test for small frequency distributions.

## Results

A total of 201 contact lens users in the Asir region, who met the inclusion criteria, were involved in the study. Table 1 shows that the respondents' ages ranged from 18 to 50 years with a mean age of 24.3 ± 12.8 years old. A total of 122 (60.7%) contact lens users had eye disease, which was myopia among 94 (77%), hyperopia among 8 (6.6%), and 20 (16.4%) had other eye diseases. As for contact lens use, 74 (36.8%) reported they use contact lenses monthly, 43 (21.4%) use lenses daily, and 36 (17.9%) use them weekly. Also, 182 (90.5%) mentioned that they wash hands before using contact lenses and make sure to change the lens solution after each use.

**Table 1:** Bio-demographic data of contact lenses user in the Asir region, Saudi Arabia.

Bio-demographic data	No.	%
Age in years		
18-30	136	67.7%
31-40	44	21.9%
41-50	21	10.4%
Previously diagnosed with eye disease		
Yes	122	60.7%
No	79	39.3%
If yes, mention		
Myopia	94	77.0%
Hyperopia	8	6.6%
Others	20	16.4%
Frequency of using lenses		
Daily	43	21.4%
Weekly	36	17.9%
Monthly	74	36.8%
Annually	48	23.9%
Do you wash your hands before using contact lenses and make sure to change the lens solution after each use?		
Yes	182	90.5%
No	19	9.5%

Table 2 reported that 91% of the study participants felt dry eye two weeks before study time, 88.1% experienced burning sensation, 82.6% complained of red eye, 80.1% felt a gritty or sandy sensation in their eyes, and 49.8% noticed crust on eyelashes. Only 41.8% got stuck shut-eye in the morning.

**Table 2:** Dry eye symptoms among contact lenses users in the Asir region, Saudi Arabia.

Dry eye symptoms	Never		Rarely		Sometimes		Often		Constantly	
	No.	%	No.	%	No.	%	No.	%	No.	%
During a typical day in the past two weeks, how often did your eyes feel dry?	18	9.0%	40	19.9%	69	34.3%	49	24.4%	25	12.4%
Do you ever feel a gritty or sandy sensation in your eyes?	40	19.9%	36	17.9%	79	39.3%	29	14.4%	17	8.5%
Do your eyes ever have a burning sensation?	24	11.9%	41	20.4%	69	34.3%	46	22.9%	21	10.4%
Are your eyes ever red?	35	17.4%	44	21.9%	59	29.4%	42	20.9%	21	10.4%
Do you notice crusting on your lashes?	101	50.2%	51	25.4%	28	13.9%	17	8.5%	4	2.0%
Do your eyes ever get stuck shut in the morning?	117	58.2%	40	19.9%	19	9.5%	18	9.0%	7	3.5%

Table 3 shows that 80.6% of the study participants felt discomfort while wearing their contact lenses. The feeling of discomfort among 30.2% was moderate in intensity (score 3) and very intense among 10.5%. Also, a feeling of dry eye was reported among 80.6%, with moderate intensity among 24.1% and very intense among 11.7%. Vision changes between clear and blurry or foggy while wearing the contact lenses were reported by 74.6% of the contact lens users with moderate intensity (score 3) among 26.7% and very intense among 8%. A total of 82.1% of the study participants reported that their eyes bothered them so much that they wanted to close them. A feeling of eye discomfort while wearing contact lenses that made them stop whatever they were doing and take out contact lenses was reported among 72.1% of the study participants. The overall CLDEQ8 score ranged from 0 to 21 with a mean score of 8.5 ± 4.9, and 57 (28.4%) users had significant CLADE.

**Table 3:** Contact lens-related effects among contact lenses users in the Asir region, Saudi Arabia. CLDEQ-8: Contact Lens Dry Eye Questionnaire-8.

CLDEQ-8		No.	%
During a typical day in the past two weeks, how often did your eyes feel discomfort while wearing your contact lenses?	Never	39	19.4%
	Rarely	46	22.9%
	Sometimes	63	31.3%
	Frequently	38	18.9%
	Constantly	15	7.5%
When your eyes felt discomfort with your contact lenses, how intense was this feeling of discomfort at the end of your wearing time?	Not at all intense	26	16.0%
	2	42	25.9%
	3	49	30.2%
	4	28	17.3%
	Very intense	17	10.5%
During a typical day in the past two weeks, how often did your eyes feel dry?	Never	39	19.4%
	Rarely	37	18.4%
	Sometimes	66	32.8%
	Frequently	43	21.4%
	Constantly	16	8.0%
When your eyes felt dry, how intense was this feeling of dryness at the end of your wearing time?	Not at all intense	34	21.0%
	2	42	25.9%
	3	39	24.1%
	4	28	17.3%
	Very intense	19	11.7%
During a typical day in the past two weeks, how often did your vision change between clear and blurry or foggy while wearing your contact lenses?	Never	51	25.4%
	Rarely	43	21.4%
	Sometimes	63	31.3%
	Frequently	34	16.9%
	Constantly	10	5.0%
When your vision was blurry, how noticeable was the changed blurry, or foggy vision at the end of your wearing time?	Not at all intense	44	29.3%
	2	29	19.3%
	3	40	26.7%
	4	25	16.7%
	Very intense	12	8.0%
During a typical day in the past two weeks, how often did your eyes bother you so much that you wanted to close them?	Never	36	17.9%
	Rarely	42	20.9%
	Sometimes	67	33.3%
	Frequently	34	16.9%
	Constantly	22	10.9%
How often during the past two weeks, did your eyes bother you so much while wearing the contact lenses you felt as if you needed to stop whatever you were doing and take out your contact lenses?	Never	56	27.9%
	Less than once a week	68	33.8%
	Weekly	18	9.0%
	Several times a week	39	19.4%
	Daily	6	3.0%
	Several times a day	14	7.0%

As for complaints during last week, a total of 66.7% of contact lens users had painful or sore eyes, 59.7% had blurred vision, 56.2% had a gritty feeling in their eyes, and 49.3% had poor vision. Regarding limited activities due to eye problems, 56.2% had problems dealing with computers or bank machines, 50.7% had problems watching TV, and 45.3% had problems in reading. Regarding the situation with an uncomfortable feeling, 62.2% felt uncomfortable in air-conditioned areas, 58.7% felt uncomfortable with windy conditions, and 51.2% felt uncomfortable in parched places, as shown in Table 4.

**Table 4:** Ocular surface disease among contact lenses users in the Asir region, Saudi Arabia. OSDI: Ocular Surface Disease Index.

OSDI	None of the time		Some of the time		Half of the time		Most of the time		All of the time	
	No.	%	No.	%	No.	%	No.	%	No.	%
Eyes that are sensitive to light	109	54.2%	58	28.9%	15	7.5%	14	7.0%	5	2.5%
Eyes that feel gritty	88	43.8%	75	37.3%	21	10.4%	13	6.5%	4	2.0%
Painful or sore eyes	67	33.3%	68	33.8%	34	16.9%	24	11.9%	8	4.0%
Blurred vision	81	40.3%	66	32.8%	28	13.9%	16	8.0%	10	5.0%
Poor vision	102	50.7%	54	26.9%	21	10.4%	14	7.0%	10	5.0%
Discomfort while reading	110	54.7%	59	29.4%	13	6.5%	11	5.5%	8	4.0%
Discomfort while driving at night	148	73.6%	34	16.9%	9	4.5%	7	3.5%	3	1.5%
Discomfort while working with a computer or bank machine (ATM)	88	43.8%	56	27.9%	29	14.4%	22	10.9%	6	3.0%
Discomfort while watching TV	99	49.3%	61	30.3%	15	7.5%	20	10.0%	6	3.0%
Discomfort during windy conditions	83	41.3%	57	28.4%	19	9.5%	24	11.9%	18	9.0%
Discomfort in places or areas with low humidity (very dry)	98	48.8%	49	24.4%	20	10.0%	21	10.4%	13	6.5%
Discomfort in areas that are air-conditioned	76	37.8%	63	31.3%	22	10.9%	19	9.5%	21	10.4%

**Figure 1:**
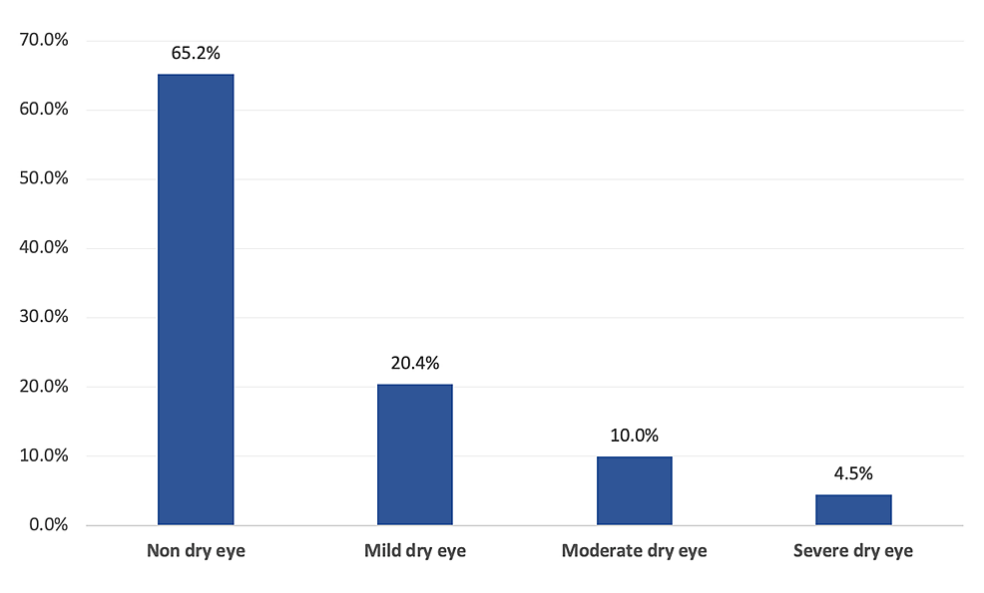
Ocular surface disease among contact lenses users in the Asir region, Saudi Arabia.

Figure 1 presents that a total of 131 (65.2%) contact lens users had non-dry eyes, 41 (20.4%) had mild dry eyes, 20 (10%) had moderate dry eyes, and 9 (4.5%) had severe dry eyes.

Table 5 shows the distribution of CLADE among contact lens users by their bio-demographic data. Dry eye was detected among 47.5% of participants diagnosed with eye disease compared to 15.2% of those who were not, with a recorded statistical significance (P = 0.001). Also, 48.8% of those who used lenses daily had dry eyes compared to 25% of annual users (P = 0.049).

**Table 5:** Distribution of contact lens-associated dry eye among lenses users by their bio-demographic data. P: Pearson's chi-squared test; $: Exact probability test; * P <0.05 (significant)

Bio-demographic data	Contact lense-associated dry eye				p-value
	Yes		No		
	No	%	No	%	
Age in years					0.263
18-30	49	36.0%	87	64.0%	
31-40	17	38.6%	27	61.4%	
41-50	4	19.0%	17	81.0%	
Previously diagnosed with eye disease					0.001*
Yes	58	47.5%	64	52.5%	
No	12	15.2%	67	84.8%	
If yes, mention					0.957^$^
Myopia	44	46.8%	50	53.2%	
Hyperopia	4	50.0%	4	50.0%	
Others	10	50.0%	10	50.0%	
Frequency of using lenses					0.049*
Daily	21	48.8%	22	51.2%	
Weekly	13	36.1%	23	63.9%	
Monthly	24	32.4%	50	67.6%	
Annually	12	25.0%	36	75.0%	
Do you wash your hands before using contact lenses and make sure to change the lens solution after each use?					0.484
Yes	62	34.1%	120	65.9%	
No	8	42.1%	11	57.9%	

## Discussion

CLD may be due to different factors, including the contact lens itself or the nearby atmosphere. The design, material, and maintenance of contact lenses are the chief factors to keep in mind. Also, patient-related factors, amenability, and ocular surface anomalies are all essentials associated with the environment. A previous study showed that dry eye symptoms were the most common complaints among contact lens wearers [15]. This is the main reason for the cessation of contact lens wear so, safety, comfort, and good visual performance should be ranked to minimize CLD and associated problems [16]. Clinically visible changes, including conjunctival indentation, conjunctival staining, conjunctival epithelial flap formation, lid wiper epitheliopathy, meibomian gland dysfunction, and Demodex blepharitis, are common features of CLD, accenting the necessity of keeping regular follow-up visits for assessment [17].

The current study aimed to assess dry eye syndrome among contact lens users. The study revealed that more than two-thirds of the contact lens users (65.2%) had non-dry eye while one-third had the disease. It was mild among one-fifth of the users (20.4%), moderate among one-tenth (10%), and 4.5% complained of severe dry eye. Dry eye disorder was significantly higher among daily contact lens users and those with a history of eye diseases. More than half of the participants used contact lenses either monthly or annually, while nearly one out of five respondents were daily users. Regarding associated symptoms, the vast majority of the wearers felt dry eyes and experienced burning sensations with red-eye. More than three-quarters (80.1%) felt a gritty or sandy sensation in their eyes, and about half of them noticed crusting on their eyelashes. A similar prevalence was assessed in a study in Al-Ahsa, Saudi Arabia. It was found that 32.1% of the population was symptomatic. Female gender, old age, smoking, and history of diabetes mellitus were found to be independent risk factors for DESs in this study [18]. Higher ocular surface disorders were a significant predictor for a higher incidence of dry eye diseases among the Saudi population [19] due to the dry and hot weather most times of the year. Alshamrani AA et al. [20] conducted a study in the eastern region of Saudi Arabia and reported that the prevalence of DES was significantly associated with female gender, old age, and history of diabetes. Other studies in Saudi Arabia found that the vast majority of the Saudi population may have some degree of DES [19,21]. Friction between the contact lens and kerato-conjunctivitis surface ends with inflammation on the ocular surface. This mostly leads to infiltration of inflammatory cells, resulting in the secretion of inflammatory cytokine and matrix metalloproteinases (MMPs). These inflammatory cytokines may cause damage in the ocular surface epithelium and then the volatility of the tear film [7,22]. Many studies estimated increased tear evaporation in contact lens wearers [23-25], but not in others. Adverse chamber trials assessed higher tear evaporation after wearing hydrogel contact lenses, but not silicon hydrogel lenses [26]. Many factors, including material, water content, and surface properties of the contact lens, could be associated with evaporation. 

Regarding the effect of CLADE syndrome, the vast majority (80.6%) of the study participants felt discomfort while wearing contact lenses, with moderate intensity (score 3) of nearly one-third of them. Additionally, a feeling of dry eyes was reported among more than three-quarters (80.6%), with moderate intensity among one-quarter of them. Three-quarters of the contact lens users reported vision changes between clear and blurry or foggy while wearing contact lenses, with moderate intensity (score 3) among 26.7%.

## Conclusions

The current study showed that nearly one out of three contact lens users in the Asir region experienced CLADE syndrome mainly with a moderate degree. On the other hand, a higher dry eye syndrome rate was demonstrated among those who reported a higher frequency of contact lens use and others with a history of eye diseases. Also, we found that DES affected different activities and daily life issues, including reading, driving, and even using computers and other screens. Contact lenses, therefore, can be a contributing factor along with other factors, but not the main or the only cause.
